# Supplementary material for: DeepCorr: a novel error correction method for 3GS long reads based on deep learning
Source: PeerJ Comput Sci. 2024 Jul 26;10:e2160. doi: 10.7717/peerj-cs.2160 (PMC11639150; doi:10.7717/peerj-cs.2160)
Supplement: Supplemental Information 2 [file peerj-cs-10-2160-s002.docx]

Supplementary files - The raw data results of various algorithms on six benchmark datasets

**Table 1.** Experimental results for the E.coli PacBio dataset.

| **Method** | **Total bases** | **Aligned bases** | **Alignment identity** | **Maximum length(bp)** | **Average length(bp)** | **N50(bp)** | **Genome**  **fraction(%)** | **User time (m:s)** | **Memory (GiB)** |
| --- | --- | --- | --- | --- | --- | --- | --- | --- | --- |
| Original | 748009625 | 729784022 | 0.9756 | 44113 | 8752 | 13990 | 100.00 | -- | -- |
| *Short-read-DBG-based methods* | | | | | | | | | |
| LoRDEC | 720464739 | 702509360 | 0.9750 | 44078 | 8435 | 13494 | 100.00 | 1194:12 | 4.8 |
| Jabba | 611947598 | 611947598 | 1.0000 | 41342 | 7880 | 12352 | 99.25 | 161:55 | 2.1 |
| *Short-read-alignment-based methods* | | | | | | | | | |
| ColorMap | 730726602 | 715441895 | 0.9790 | 44113 | 8529 | 13641 | 100.00 | 1728:22 | 24.5 |
| Proovread | 537183316 | 537132179 | 0.9999 | 39836 | 4971 | 9435 | 100.00 | 2804:4 | 7.5 |
| *HMM-based method* | | | | | | | | | |
| Hercules | 742217998 | 723994674 | 0.9754 | 44113 | 8691 | 13887 | 100.00 | 95964:55 | 4.8 |
| *Self-correction method* | | | | | | | | | |
| VeChat | 600124585 | 597111612 | 0.9949 | 40972 | 9108 | 13695 | 100.00 | 9358:3 | >50 |
| *Deep learning-based methods* | | | | | | | | | |
| DeepCorr | 748417338 | 748015166 | 0.9995 | 44113 | 8768 | 14020 | 100.00 | 650:50 | 3.4 |

**Table 2.** Experimental results for the E.coli ONT dataset.

| **Method** | **Total bases** | **Aligned bases** | **Alignment identity** | **Maximum length(bp)** | **Average length(bp)** | **N50(bp)** | **Genome**  **fraction(%)** | **User time (m:s)** | **Memory (GiB)** |
| --- | --- | --- | --- | --- | --- | --- | --- | --- | --- |
| Original | 1481511788 | 1479176967 | 0.9984 | 131969 | 9047 | 14895 | 100.00 | -- | -- |
| *Short-read-DBG-based methods* | | | | | | | | | |
| LoRDEC | 1553433548 | 1552247152 | 0.9992 | 137934 | 9481 | 15653 | 100.00 | 2960:20 | 4.8 |
| Jabba | 1258239439 | 1258239439 | 1.0000 | 93396 | 7709 | 12436 | 99.52 | 137:49 | 2.1 |
| *Short-read-alignment-based methods* | | | | | | | | | |
| ColorMap | 1518333301 | 1516962292 | 0.9990 | 134311 | 9253 | 15180 | 100.00 | 1811:59 | 22.7 |
| Proovread | 979107621 | 979107621 | 1.0000 | 28387 | 1378 | 1662 | 100.00 | 9765:59 | 7.5 |
| *HMM-based methods* | | | | | | | | | |
| Hercules | 1488092513 | 1485766466 | 0.9984 | 132948 | 9087 | 14974 | 100.00 | 136645:53 | 7.0 |
| *Self-correction method* | | | | | | | | | |
| VeChat | 1013354131 | 1013331827 | 0.9999 | 129860 | 10282 | 16010 | 99.99 | 21878:17 | >50 |
| *Deep learning-based methods* | | | | | | | | | |
| DeepCorr | 1480818530 | 1480547290 | 0.9998 | 132220 | 9055 | 14918 | 100.00 | 947:2 | 3.4 |

**Table 3.** Experimental results for the Yeast PacBio dataset.

| **Method** | **Total bases** | **Aligned bases** | **Alignment identity** | **Maximum length(bp)** | **Average length(bp)** | **N50(bp)** | **Genome**  **fraction(%)** | **User time (m:s)** | **Memory (GiB)** |
| --- | --- | --- | --- | --- | --- | --- | --- | --- | --- |
| Original | 5499119594 | 4853379662 | 0.8825 | 94868 | 9108 | 18406 | 99.97 | -- | -- |
| *Short-read-DBG-based methods* | | | | | | | | | |
| LoRDEC | 5375583040 | 4749661372 | 0.8835 | 94868 | 8910 | 17991 | 99.99 | 4282:28 | 4.8 |
| Jabba | 2192986588 | 2183714060 | 0.9957 | 46975 | 8501 | 12780 | 95.25 | 990:47 | 2.1 |
| *Short-read-alignment-based methods* | | | | | | | | | |
| ColorMap | 5506697225 | 4860976804 | 0.8827 | 94868 | 9120 | 18434 | 99.97 | 3450:20 | 6.5 |
| Proovread | -- | -- | -- | -- | -- | -- | -- | -- | -- |
| *HMM-based method* | | | | | | | | | |
| Hercules | 5494486747 | 4848765700 | 0.8824 | 94868 | 9102 | 18392 | 99.97 | 202667:27 | 5.0 |
| *Self-correction method* | | | | | | | | | |
| VeChat | 1396690725 | 1395321446 | 0.9990 | 50917 | 12801 | 18394 | 99.97 | 48860:39 | >50 |
| *Deep learning-based methods* | | | | | | | | | |
| DeepCorr | 5602620449 | 5388605716 | 0.9618 | 94868 | 9084 | 18459 | 99.97 | 2193:16 | 3.4 |

Note: When using the default parameters provided by Proovread for error correction on this dataset, the algorithm reports a warning: “Duplicated reads” and terminates the correction process automatically. Therefore, the experimental results of Proovread on this table are “--”.

**Table 4.** Experimental results for the Yeast ONT dataset.

| **Method** | **Total bases** | **Aligned bases** | **Alignment identity** | **Maximum length(bp)** | **Average length(bp)** | **N50(bp)** | **Genome**  **fraction(%)** | **User time (m:s)** | **Memory (GiB)** |
| --- | --- | --- | --- | --- | --- | --- | --- | --- | --- |
| Original | 382389287 | 376989685 | 0.9858 | 56477 | 9186 | 11696 | 99.97 | -- | -- |
| *Short-read-DBG-based methods* | | | | | | | | | |
| LoRDEC | 390946752 | 386159193 | 0.9877 | 58334 | 9391 | 11971 | 99.99 | 473:10 | 4.8 |
| Jabba | 288736216 | 288726754 | 0.9999 | 47266 | 7993 | 10719 | 94.75 | 115:3 | 2.1 |
| *Short-read-alignment-based methods* | | | | | | | | | |
| ColorMap | 385129056 | 379728102 | 0.9859 | 56785 | 9245 | 11775 | 99.97 | 1461:6 | 2.4 |
| Proovread | 100640 | 90149 | 0.8957 | 1102 | 602 | 599 | 1.29 | 513:27 | 7.5 |
| *HMM-based method* | | | | | | | | | |
| Hercules | 383933798 | 378533253 | 0.9859 | 57481 | 9223 | 11748 | 99.97 | 64005:15 | 3.2 |
| *Self-correction method* | | | | | | | | | |
| VeChat | 344942675 | 343633076 | 0.9962 | 56929 | 9507 | 11648 | 99.19 | 18931:54 | >50 |
| *Deep learning-based methods* | | | | | | | | | |
| DeepCorr | 383386288 | 383198354 | 0.9995 | 56828 | 9221 | 11747 | 99.97 | 271:8 | 3.4 |

**Table 5.** Experimental results for the Fruit fly PacBio dataset.

| **Method** | **Total bases** | **Aligned bases** | **Alignment identity** | **Maximum length(bp)** | **Average length(bp)** | **N50(bp)** | **Genome**  **fraction(%)** | **User time (m:s)** | **Memory (GiB)** |
| --- | --- | --- | --- | --- | --- | --- | --- | --- | --- |
| Original | 277577924 | 163084772 | 0.5875 | 54186 | 2371 | 12627 | 58.48 | -- | -- |
| *Short-read-DBG-based methods* | | | | | | | | | |
| LoRDEC | 273502325 | 159557680 | 0.5833 | 54186 | 2337 | 12294 | **59.75** | 510:42 | 4.8 |
| Jabba | 68570230 | 68562572 | 0.9998 | 31567 | 4980 | 7700 | 35.75 | 2142:49 | 2.1 |
| *Short-read-alignment-based methods* | | | | | | | | | |
| ColorMap | 275086625 | 168238015 | 0.6115 | 53985 | 2349 | 12369 | 61.08 | 6139:10 | 2.4 |
| Proovread | 132095964 | 132095964 | 0.5911 | 30412 | 4600 | 8617 | 59.32 | 4102:13 | 7.5 |
| *HMM-based methods* | | | | | | | | | |
| Hercules | 275620916 | 162943476 | 0.5912 | 54186 | 2354 | 12420 | 58.88 | 21157:17 | 3.2 |
| *Self-correction method* | | | | | | | | | |
| VeChat | 35321050 | 34762504 | 0.9841 | 46655 | 12792 | 17048 | 13.564 | 270:47 | >50 |
| *Deep learning-based methods* | | | | | | | | | |
| DeepCorr | **279293657** | **192652283** | **0.6898** | **54186** | **2386** | **12839** | 57.76 | **256:26** | **3.4** |

**Table 6.** Experimental results for the Fruit fly ONT dataset.

| **Method** | **Total bases** | **Aligned bases** | **Alignment identity** | **Maximum length(bp)** | **Average length(bp)** | **N50(bp)** | **Genome**  **fraction(%)** | **User time (m:s)** | **Memory (GiB)** |
| --- | --- | --- | --- | --- | --- | --- | --- | --- | --- |
| Original | 4609479994 | 4193853794 | 0.9098 | 446050 | 7177 | 11956 | 99.59 | -- | -- |
| *Short-read-DBG-based methods* | | | | | | | | | |
| LoRDEC | **4663484804** | 4272461697 | 0.9161 | **467983** | **7253** | **12105** | 99.58 | 11130:43 | 4.8 |
| Jabba | 2277474552 | 2277352751 | 0.9999 | 47190 | 3962 | 6081 | 84.84 | 11999:4 | 2.1 |
| *Short-read-alignment-based methods* | | | | | | | | | |
| ColorMap | 4685641775 | 4290298819 | 0.9156 | 444791 | 7226 | 12046 | 99.60 | **5338:54** | **2.4** |
| Proovread | -- | -- | -- | -- | -- | -- | -- | -- | -- |
| *HMM-based methods* | | | | | | | | | |
| Hercules | 4615570873 | 4202349986 | 0.9104 | 446013 | 7185 | 11974 | 99.59 | 198745:3 | 3.2 |
| *Self-correction method* | | | | | | | | | |
| VeChat | 2589870493 | 2587909418 | 0.9992 | 412297 | 8271 | 12513 | 99.31 | 110678:22 | >50 |
| *Deep learning-based methods* | | | | | | | | | |
| DeepCorr | 4613233549 | **4567219125** | **0.9900** | 446068 | 7189 | 11982 | **99.60** | 7631:58 | 3.4 |

Note: The correction process of Proovread on this dataset exceeds 15 days on a 72-core computer and the iteration goes on. Consequently, the correction process is terminated manually, and the experimental results are marked with “--”.
